# Medwakh smoking induces alterations in salivary proteins and cytokine expression: a clinical exploratory proteomics investigation

**DOI:** 10.1186/s12014-024-09520-6

**Published:** 2025-01-17

**Authors:** K. G. Aghila Rani, Nelson C. Soares, Betul Rahman, Alexander D. Giddey, Hamza M. Al-Hroub, Mohammad H. Semreen, Sausan Al Kawas

**Affiliations:** 1 Research Institute for Medical and Health Sciences, University of Sharjah, P.O. Box 27272, Sharjah, UAE; 2 Center for Applied and Translational Genomics (CATG), Mohammed Bin Rashid University of Medicine and Health Sciences, Dubai Health, Dubai, UAE; 3 College of Medine, Mohammed bin Rashid Al Maktoum University of Medicine and Health Sciences, Dubai Health, Dubai, UAE; 4 Department of Human Genetics, National Institute of Health Doutor Ricardo Jorge (INSA), Lisbon, Portugal; 5 Department of Preventive and Restorative Dentistry, College of Dental Medicine, University of Sharjah, Sharjah, UAE; 6 Department of Oral and Craniofacial Health Sciences, College of Dental Medicine, University of Sharjah, Sharjah, UAE

**Keywords:** Medwakh smoking, salivary proteomics, salivary immune response, involucrin, oxidative stress, cell metabolism

## Abstract

**Background:**

Medwakh smoking has radically expanded among youth in the Middle East and around the world. The rising popularity of medwakh/dokha usage is linked to the onset of several chronic illnesses including cardiovascular diseases and cancers. Medwakh smoking is reported to increase the risk of inflammation in the lower respiratory tract owing to oxidative burden. To date, there are no reported studies investigating the impact of medwakh smoking on salivary protein profile. The current study aims to elucidate alterations in the salivary proteome profile of medwakh smokers.

**Methods:**

Saliva samples collected from 33 medwakh smokers and 30 non-smokers were subjected to proteomic analysis using UHPLC-ESI-QTOF-MS. Saliva samples were further subjected to validatory experiments involving analysis of inflammatory cytokine profile using LEGENDplex™ Human Essential Immune Response Panel.

**Results:**

Statistical analysis revealed alterations in the abundance of 74 key proteins including immune mediators and inflammatory markers in medwakh smokers (Accession: PXD045901). Proteins involved in building oxidative stress, alterations in cell anchorage, and cell metabolic processes were enhanced in medwakh smokers. Salivary immune response evaluation further validated the proteome findings, revealing significantly higher levels of IL-1β, IL-12p70, IL-23, IFN-γ (Th1 cytokines), IL-6 (Th2 cytokine), and MCP-1 (chemokine) in medwakh smokers. In addition, a substantial increase in abundance of involucrin suggesting a plausible stratified squamous cell differentiation and increased cell lysis in the oral cavity of medwakh smokers akin to chronic obstructive pulmonary diseases (COPD). The protein–metabolite joint pathway analysis further showed significantly enriched differentially expressed proteins and metabolites of glycolysis/gluconeogenesis, pentose phosphate, fructose and mannose, nicotinate and nicotinamide, and glutathione metabolism pathways among medwakh smokers.

**Conclusions:**

The findings of the study provide valuable insights on potential perturbations in various key immune molecules, cytokines, and signaling pathways among medwakh smokers. Medwakh smokers displayed elevated inflammation, increased oxidative stress and defective antioxidant responses, dysregulated energy metabolism, and alterations in proteins related to cell adhesion, migration, differentiation, and proliferation. The findings of study underscore the urgent need for comprehensive public health interventions among youth by raising awareness, implementing effective smoking cessation programs, and promoting healthy lifestyle to safeguard the well-being of individuals and communities worldwide.

**Supplementary Information:**

The online version contains supplementary material available at 10.1186/s12014-024-09520-6.

## Background

Tobacco smoking has a strong link to the onset of various cancers, cardiovascular diseases, stroke, and respiratory illnesses [1, 2]. Tobacco kills more than 7, 000, 000 individuals every year, globally [3]. Additionally, the consumption of alternative tobacco products (ATP) such as medwakh/dokha has experienced a significant surge, particularly among youth, including schoolchildren, college students, and the general adult population, over the past decade [4]. Medwakh smokers are at a higher risk of nicotine addiction compared to cigarette smokers or users of other ATP products. Reported nicotine levels in various brands/forms of medwakh/dokha range from 23.82 to 52.8 mg/g. Despite FDA warnings, its popularity among teenagers is increasing and largely perceived as a safer alternative to cigarettes. Moreover, medwakh smokers are more prone to severe oral infections and are 7 to 10 times more likely to develop oral cancers than non-smokers [5]. Additionally, medwakh smoking increases the risk of inflammatory conditions in the lower respiratory tract through multiple interrelated mechanisms, with oxidative stress being a significant contributing factor [6].

Increased oxidative stress and the consequent release of cytokines or chemokines are commonly linked to the progressive accumulation of reactive oxygen species (ROS) in oral fluids and tissues [7]. Previous research has documented the presence of markers of lipid peroxidation, protein oxidation, and DNA damage in the saliva of traditional smokers [8]. These oxidative stress markers contribute to an imbalance in the antioxidant defense system of saliva [9]. A compromised antioxidant defense renders oral epithelial cells susceptible to damage from thiocyanate ions and hydroxyl free radicals present in tobacco smoke. This susceptibility frequently results in the development of oral cancers.

Cell damage in oral tissues leaves distinct molecular and protein signatures in saliva, which are increasingly utilized for non-invasive early detection of tobacco-related oral diseases. Integrating data from proteomics and metabolomics holds promise for a deeper understanding of the pathophysiology associated with various diseases [10]. In a comparative salivary metabolomics and proteomics study, alterations in the abundance of interleukin-1 receptor antagonists, thioredoxin, and lipocalin-1 were observed in heavy cigarette smokers [11]. The release of toxic components from tobacco smoke disrupts cellular physiology, metabolic processes, immune responses, inflammatory pathways in oral tissues, and antioxidant defenses in saliva, contributing to the eventual development of chronic obstructive pulmonary disease (COPD). However, to date, there are no reported studies specifically investigating the toxic effects of medwakh smoking on salivary proteins.

In a previous study, we conducted a comprehensive salivary metabolomics analysis of medwakh smokers, establishing their strong association with redox homeostasis and inflammation [6]. The current study further elucidated the molecular landscape in medwakh smoker’s saliva and corroborated with the metabolomics findings, providing deeper insights into specific dysregulated proteins with a focus on markers of immunity and inflammation. Other factors we assessed include oxidative stress, cellular-level alterations and their associations with prognostic factors associated with COPD and oral cancers. By integrating metabolomics and proteomics analysis by a joint pathway analysis, we further validate and expand knowledge on early-stage biomarkers indicative of the risk of tobacco-related diseases among medwakh. To our knowledge, there are no existing proteomics-based studies identifying salivary biomarkers in medwakh smokers.

## Methods

### Study design and participants’ characteristics

The present study utilized the same cohort as our previous research on the salivary metabolomics with three additional medwakh smoker samples [6]. The current study included 33 medwakh smokers (median age, IQR; 24 (3) and 30 non-smokers (median age, IQR; 23.5(1).

Ethical approvalfor the study procedure was obtained from the University of Sharjah’s Research Ethics Committee (REC-18-10-23-01), adhering to national and international guidelines, including the Helsinki Declaration. Participants were provided with detailed information about the study design and objectives, and written informed consent was obtained from each participant before sample collection commenced. Inclusion criteria encompassed young individuals who exclusively smoked medwakh, without using any other tobacco products, as well as individuals who had never smoked.

Participants undergoing any orthodontic treatments and those receiving periodontal therapies, antibiotics, or steroid therapies throughout the trial or within three months of study frame were not included in the study. Participants with chronic inflammatory disorders or other medical complications, were excluded from the study. In addition, all participants were men, as they constituted the majority of medwakh smokers. Each participant was asked to complete a questionnaire detailing demographic information, overall health status, periodontal condition, frequency of medwakh use, and any history of smoking other tobacco products.

Unstimulated saliva samples were collected from the study participants in sterile collection tubes. To avoid sample contamination, participants were advised to observe a two-hour fast during which they were not allowed to eat, drink, or smoke. The samples were centrifuged for 5 min at 4 °C at 2500 rpm to remove cell debris, and the supernatants were then kept at 80 °C for analysis. Statistical analysis involved conducting chi-square tests and Mann-Whitney tests to compare participant characteristics and age between medwakh smokers and non-smokers, respectively.

### Chemicals and reagents

Methanol (≥ 99.9%), acetonitrile (ACN) and deionized water, as well as LC-MS CHROMASOLV, were purchased from Honeywell (Seelze, Germany). Formic acid (FA) and trifluoroacetic acid (TFA) were purchased from Fisher Scientific (Loughborough, UK). Dithiothreitol (DTT), iodoacetamide (IAA), pierce protease inhibitor tablets, pierce trypsin protease, IP-lysis buffer and Lysl endopeptidase (Lys-C) were purchased from Thermo Scientific (Rockford, USA). Bradford’s reagent was purchased from Sigma-Aldrich (St. Louis, USA).

### Sample protein extraction and quantification

150 µl of saliva was mixed with 400 µL IP-lysis buffer containing 1x protease inhibitor and incubated for 10 min at room temperature. The sample lysates were sonicated at 30% amplification for 30 s followed by centrifugation at 15,000 rpm at 4 °C for 5 min. The supernatants were collected, and protein precipitation was performed by the addition of 400 µL methanol and 300 µL chloroform. The samples were mixed thoroughly and centrifuged at 13,000 rpm at 4 °C for 5 min. The upper aqueous phase was discarded, and the lower phase along with the white protein disks was mixed with 300 µL of methanol. The samples were further centrifuged at 13,000 rpm at 4 °C for 1 min and the supernatants were discarded. The protein pellets were further air-dried and resuspended in 100 µL of denaturation solution (6 M urea, 2 M thiourea in 10mM Tris buffer, pH 8), and quantified using a modified Bradford assay [12]. This method allowed consistent recovery of more than 100 µg of protein as estimated by the Bradford protein assay.

### Protein digestion and peptide purification

Protein samples were prepared for LC-MS/MS analysis by in-solution digestion [13]. Briefly, 100 µg of protein from all samples in denaturation buffer at a final volume of 100 µL were reduced with 10 µL of 10 mM dithiothreitol (DTT) (final concentration 1 mM) for 1 h with gentle agitation at 100 rpm and room temperature (RT). Samples were then alkylated using 10 µL of 55 mM iodoacetamide (IAA) (final concentration 5.5 mM) and incubated for 1 h in the dark at 100 rpm at RT. The pH was adjusted to 8.0 in all these reactions. Protein digestion was performed using 1 µg of Lysyl Endopeptidase LysC (1:100 ratio) and samples were incubated for 3 h at 100 rpm at RT followed by dilution in 4 volumes of 20 mM ammonium bicarbonate and 1 µg of trypsin digestion (1:100 ratio). The samples were then subjected to overnight incubation at 100 rpm in RT. Then the samples were dried using EZ-2 Plus (GeneVac-Ipswich, UK) and resuspended with 100 µL of 1% trifluoroacetic acid (TFA).

Peptides were then desalted using Pierce^®^ C18 stage tips from Thermo Scientific (Waltham, USA). Pierce^®^ C 18 tips were activated by repeatedly aspirating and dispensing 100 µL of 50% ACN, subsequently equilibrating with 100 µL of 0.1% trifluoroacetic acid (TFA), followed by loading with sample peptides by repeatedly aspirating and dispensing the sample volume (10 times). Similarly, the peptides were washed with 5% ACN, 0.1% TFA, and eluted by gently aspirating and dispensing 100 µl of 60% ACN, 0.1% formic acid (FA) repeatedly ten times. Before LC-MS/MS analysis, desalted peptides were dried in EZ-2 Plus and resolubilized in 2% ACN, and 0.1% FA.

### Nanoflow High-pressure Liquid Chromatography-Tandem Mass Spectrometry (nano-HPLC-MS/MS)

The LC-MS/MS analysis was carried out by using a nano elute in conjunction with a quadrupole-time-of-flight mass spectrometer (Q-TOF) and a CaptiveSpray ion source (Bruker Daltonics, Bremen, Germany). The PC used Windows 10 Enterprise 2016 LTSB as its operating system. Bruker Compass HyStar 5.0 SR1 Patch1 (5.0.37.1), Compass 4.1 for otofSeries, and otof Control Version 6.2 served as the data management software.

Thermo Fisher Scientific’s Acclaim PepMap C18 trap cartridge, which has a 5 mm, 300 μm id, 5 μm particle diameter, and 100 Å pore size was used to capture the injected peptides in a 4 µl aliquot of each sample (4 µg of the peptides). On a FIFTEEN column C18 15 cm x 75 μm, 1.9 μm (Bruker), sample separation was carried out using solvents A and B with a 140-min gradient as follows: 0 to 5 min, 5% B; 5 to 120 min, 5–35% B; 120 to 125 min, 35–95% B; 125 to 135 min, 95% B; 135 to 135.2 min, 95 − 5% B; 135.2–140, 5% B. 0.1% formic acid in deionized Water and 0.1% formic acid in acetonitrile was used as solvents A and B respectively. The flow rate maintained was 0.30 µL/min.

The drying gas flow rate was 3.0 L/min at a temperature of 150 °C and the capillary voltage was set at 1600 V for each injection of the Captive Spray ion source. Fragmentation was a part of the auto MS/MS scan with CID acquisition that was used for the acquisition. The acquisition was carried out at 2 Hz in positive mode. The automatic in-run mass scan range was from 150 to 2200 m/z. The width of the precursor ion, cycle time, and threshold were ± 0.5, 3.0 s, and 500 cts respectively. The active exclusion was triggered after 1 spectrum and was released after 0.4 min.

Data-dependent acquisition (DDA) was employed for MS2 acquisition, and the collision energy was modified from 23 to 65 eV as a function of the m/z value. The m/z measurements were externally calibrated using 10 mM of sodium formate before sample analysis. In addition, sodium formate solution was injected every (3–4 samples) to check the accuracy of m/z. Also, Pierce HeLa protein digest standard (Thermo Scientific) was injected before starting batch analysis and used as a quality control sample for mass spectrometry (MS) analysis of proteomic samples.

### Cytometric bead array for the estimation of cytokine levels in the saliva samples

To further validate the findings observed in the proteomics data, cytokine bead array was performed to determine the levels of the cytokine sin the saliva of medwakh smokers. LEGENDplex™ Human Essential Immune Response Panel (13-plex) Biolegend, San Diego, USA) was used for the detection of cytokines in saliva samples following manufacturer’s instructions. Briefly, 33 µl of both nonsmoker and medwakh smoker saliva samples were mixed with 25 µl of assay buffer and microbeads each and incubated for 2 h in dark in mild rotation at 800 rpm. The samples were washed with wash buffer with centrifugation at 1300 rpm for 5 min followed by incubation with 25 µl of detection antibodies for 60 min and 25 µl of SA-PE for an additional 30 min. Samples were further washed in 1x wash buffer, suspended in 150 µl of buffer and acquired on a cytoflex flow cytometer. Analysis was performed using LEGENDplex™ data analysis software.

### Data analysis and statistical testing

The Uniprot proteome for Homo sapiens (Proteome ID: UP000005640, 75,776 entries, 11/09/2021) and the Andromeda search engine were used to identify proteins and peptides in the raw data using the MaxQuant version 1.6.17.0. The default parameter settings employed for the MS/MS database search include acetylation of protein N-termini and methionine oxidation as the variable modifications and carbamidomethylation of cysteine residues as the fixed modification. Filtered peptide spectral matching (PSMs) had a false discovery rate (FDR) of 1% and a precursor mass tolerance of 20 ppm. For in silico digestion, the trypsin/P enzymatic cleavage algorithm was applied, and for label-free quantitation (LFQ), the MaxLFQ method was employed.

A custom R script was used for downstream analysis of the proteomics data. Data was filtered to remove hits to the reverse database and potential contaminants, as well as to remove proteins not present in at least 70% of the samples of at least one group. Missing data was imputed with half the minimum value observed on a per-protein basis and sample clustering was visualised by unsupervised Principal Component Analysis. Differentially abundant proteins were defined as those with a Benjamini-Hochberg adjusted p-value of < 0.05. Volcano plots and boxplots for differentially abundant proteins were visualised using the R package ggplot2. We employed the R package ‘clusterProfiler’ as well as Reactome (https://reactome.org/) for pathway (as per the Kyoto Encyclopedia of Genes and Genomes, KEGG) and Gene Ontology (GO) term enrichment analysis by way of over-representation analysis for those proteins that were significantly differentially abundant, requiring an adjusted p-value < 0.05 for the pathway/GO term enrichment. Quantitative assessments of cytokine expression was performed using Graphpad prism (version 9.1), and statistical differences among non-smokers and medwakh smokers for the expression of pro- and anti-inflammatory cytokines and chemokines were calculated by Mann–Whitney U tests. Statistical tests were two-tailed with p < 0.05 considered significant.

## Results

### Study participants

The study participants included 33 medwakh smokers and 30 non-smokers. The medwakh group smoked more than five times a day on average. All the participants were young adults with ages ranging from 19 to 25. The median age of medwakh smokers and nonsmokers were 24 (3) and 23.5 (1) respectively. Except for smoking history (*p* < 0.001) and use of interdental aids (*p* < 0.05), no significant correlation was observed in participant characteristics and age by statistical analysis performed by chi-square test and Mann-Whitney test respectively between medwakh smokers and non-smokers. Similarly, we did not find any significant association when the median age of medwakh smokers (24) and non-smokers (23.5) were compared (*p* = 0.851); Table 1 [6]).

**Table 1:** Demographics of the study participants divided into two groups based on smoking status [6]

Characteristics	Categories	Medwakh		Non-smoker		*p*-value
		*n*	%	*n*	%	
Age	Median, IQR	24 (3)		23.5 (1)		0.851
Education level	Intermediate	1	3.3%	0	0.0%	0.313
	High (university degree)	31	96.7%	30	100.0%	
Smoking history	Yes	32	96.7%	0	0.0%	< 0.001
	No	1	3.3%	30	100.0%	
Tooth brushing frequency	At least twice daily	19	63.3%	20	66.7%	0.962
	Once a day	10	33.3%	9	30.0%	
	Less frequently	4	3.3%	1	3.3%	
Interdental aid use	Daily	9	20.0%	15	50.0%	0.037
	Sometimes	8	26.7%	7	23.3%	
	Never	16	53.3%	8	26.7%	

### Salivary proteome profile of Medwakh smokers showed significant alterations

Data are available via ProteomeXchange with identifier PXD045901. In the comparative proteomics analysis, 4.7 × 10^6^ spectra were acquired, and 460,873 spectra were identified, yielding 7590 non-redundant peptides, 6672 of which were unique to protein groups identified across 63 saliva samples. Tryptic cleavage efficiency was acceptable with 64% of the identified peptides having no missed cleavage sites (supplementary Fig. 1). The peptide charge distribution revealed a predominance of double and triple charged species as predicted. Using a decoy database strategy and allowing for at most 1% false discovery rate (FDR) at both the peptide and the protein level, MaxQuant analysis of the combined LC-MS/MS data yielded 749 unique protein groups with a median of 6 assigned (minimum 2) and 5 unique (minimum 1) peptides per protein group. Of the 749 protein groups, 422 were retained for quantitative statistical testing after applying a 70% valid value cut-off filter, in which a quantitative value was required for at least 70% of the samples of at least one of the two groups in order to be retained. Of these 422 proteins, 131 were observed to be differentially abundant by t-test (Benjamini-Hochberg multiple testing correction) with a p-value of < 0.05, between the two study groups (supplementary table S1). No samples were omitted and all the study samples (medwakh smoker: *N* = 33 and non-smoker: *N* = 30) were considered for the analysis. Protein identification table generated by Maxquant is given in supplementary table S1.

### Differential clustering of the salivary proteome profile of medwakh smokers

**Fig. 1:**
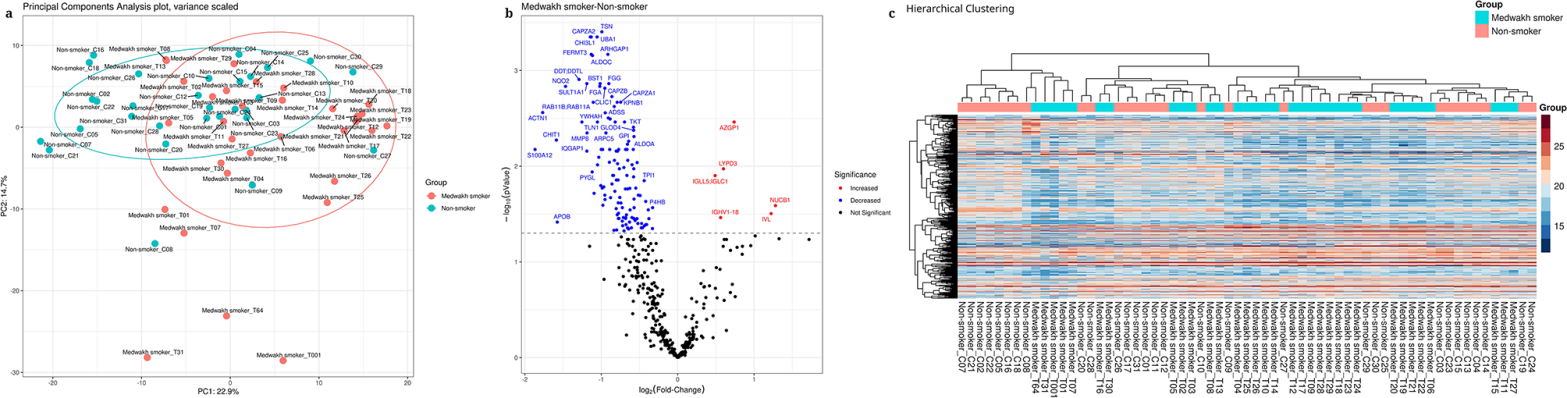
(**a**) Principal component analysis plot of medwakh smokers along with non-smokers. The distribution of proteome profiles of medwakh smokers (green, *n* = 33) and non-smokers (red, *n* = 30) are displayed 2-dimensionally based on smoking status. A well-defined distribution in the proteome profile was observed in medwakh smokers in comparison to non-smokers. (**b**) Volcano plot displaying proteins that altered significantly in medwakh smokers vs. non-smokers. Log2 fold-changed proteins (medwakh smoker/non-smoker) plotted against the -log10 (p-value) highlighting the differentially abundant proteins (two-sample t-test with BH FDR < 0.05). (**c**) Heatmap with hierarchical clustering of proteomics profile showing relative quantified proteins in the saliva samples of medwakh smokers and non-smokers

Principal component analysis (PCA) showed a clear separation in the protein profiles of the study groups based on the smoking status. A well-defined distribution of the proteome profile was observed among medwakh smokers (Fig. 1a). A volcano Plot was further constructed to graphically represent the results of the t-test for the differential level of the unique proteins. Interestingly, among these differentially significant abundant proteins, we found an enhanced abundance for 6 proteins in medwakh smokers and the remaining were significantly less abundant (*p* < 0.05 and log2 (fold-change) > 1); Table 2; Fig. 1b). Further, hierarchical clustering and heatmap visualization presented more noticeable changes due to medwakh smoking at the protein level. Heat map analysis of the most significantly abundant proteins affected by medwakh smoking revealed the top-ranking proteins as shown in Fig. 1c. Immunoglobulin lambda like polypeptide 5 (IGLL5), Immunoglobulin heavy variable 1–18 (IGHV1-18), Ly6/PLAUR domain-containing protein 3 (LYPD3), Zinc-alpha-2-glycoprotein (AZGP1) and Nucleobindin-1 (NUCB1) were significantly abundant in medwakh smokers. The highly reactive and soluble transglutaminase substrate protein, involucrin (IVL), expressed by keratinocytes of the epidermis was also found to be significantly abundant among the medwakh smoking group. Among the altered key proteins includes those playing key roles in the regulation of immune complex formation (complement factor I, chitinase-3-like protein 1), hemostasis, thrombosis, and oxidative burst (chloride intercellular channel protein 1, neutrophil cytosol factor 4), cell anchorage (F-actin-capping protein subunit alpha-2, Talin-1), nucleotide metabolism (adenylosuccinate synthetase isozyme 2), cellular metabolism (glucose-6-phosphate 1-dehydrogenase, hexokinase-3), pathogen clearance several glycoproteins (proteasome activator complex subunit 2, protein S100-A12, histidine-rich glycoprotein), and cellular proteins degradation (Ubiquitin-like modifier-activating enzyme 1) Table 2).

**Table 2:** List of significantly differential abundant proteins highlighting the impact of medwakh smoking on cellular structure and function, inflammatory pathways, oxidative stress, antioxidant responses, and cellular metabolic events in comparison to non-smokers

Protein ID	Protein name				
**Cell anchorage, adhesion and cytoskeletal motility**					
FERMT3	Fermitin family homolog 3				↓
CAPZA2	F-actin-capping protein subunit alpha-2				↓
CAPZA1 F	F-actin-capping protein subunit alpha-1				↓
ARPC4	Actin-related protein 2/3 complex subunit 4				↓
CPPED1	Serine/threonine-protein phosphatase				↓
EEF1G	Elongation factor 1-gamma				↓
CAPZA2	F-actin-capping protein subunit alpha-2				↓
ARPC2	Actin-related protein 2/3 complex subunit 2				↓
CPPED1	Serine/threonine-protein phosphatase				↓
EEF1G	Elongation factor 1-gamma				↓
SPTAN1	Spectrin alpha chain, non-erythrocytic 1				↓
ARPC5	Actin-related protein 2/3 complex subunit 5				↓
ACTN1	Alpha-actinin-1				↓
TLN1	Talin-1				↓
IVL	Involucrin				↑
LYPD3	Ly6/PLAUR domain-containing protein 3				↑
AZGP1	Zinc-alpha-2-glycoprotein				↑
**Immune responses and inflammation**					
PSME2	Proteasome activator complex subunit 2				↓
CFI	Complement factor I				↓
S100A12	Protein S100-A12				↓
CHI3L1	Chitinase-3-like protein 1				↓
BST1	ADP-ribosyl cyclase/cyclic ADP-ribose hydrolase 2				↓
HRG	Histidine-rich glycoprotein				↓
RNASE2	Non-secretory ribonuclease				↓
MNDA	Myeloid cell nuclear differentiation antigen				↓
ALDOC	Fructose-bisphosphate aldolase C				↓
IGLL5	Immunoglobulin lambda like polypeptide 5				↑
IGHV1-18	Immunoglobulin heavy variable 1–18				↑
**Oxidative stress responses**					
CLIC1	Chloride intracellular channel protein 1				↓
NCF4	Neutrophil cytosol factor 4				↓
NAPRT	Nicotinate phosphoribosyltransferase				↓
ERP29	Endoplasmic reticulum resident protein 29				↓
AKR1A1	Aldo-keto reductase family 1 member A1				↓
BLVRB	Flavin reductase (NADPH)				↓
**Antioxidant responses**					
NCF2	Neutrophil cytosol factor 2				↓
NCF1	Neutrophil cytosol factor 1				↓
NCF1C	Putative neutrophil cytosol factor 1 C				↓
NCF1B	Putative neutrophil cytosol factor 1B				↓
AKR1A1	Aldo-keto reductase family 1 member A1				↓
**Cell metabolism**					↓
G6PD	Glucose-6-phosphate 1-dehydrogenase				↓
GYG1	Glycogenin-1				↓
HK3	Hexokinase-3				↓
PYGL	Glycogen phosphorylase				↓
UGP2	UTP–glucose-1-phosphate uridylyltransferase				↓
NUCB1	Nucleobindin-1				↑
**Unique proteins**					
ADSS	Adenylosuccinate synthetase isozyme 2				↓
ARHGAP1	Rho GTPase-activating protein 1				↓
B4GAT1	Beta-1,4-glucuronyltransferase 1				↓
FGA	Fibrinogen alpha chain				↓
NQO2	Ribosyldihydronicotinamide dehydrogenase [quinone]				↓
SULT1A1	Sulfotransferase 1A1				↓
TSN	Translin				↓
UBA1	Ubiquitin-like modifier-activating enzyme 1				↓

### Highlights of the study

**Fig. 2:**
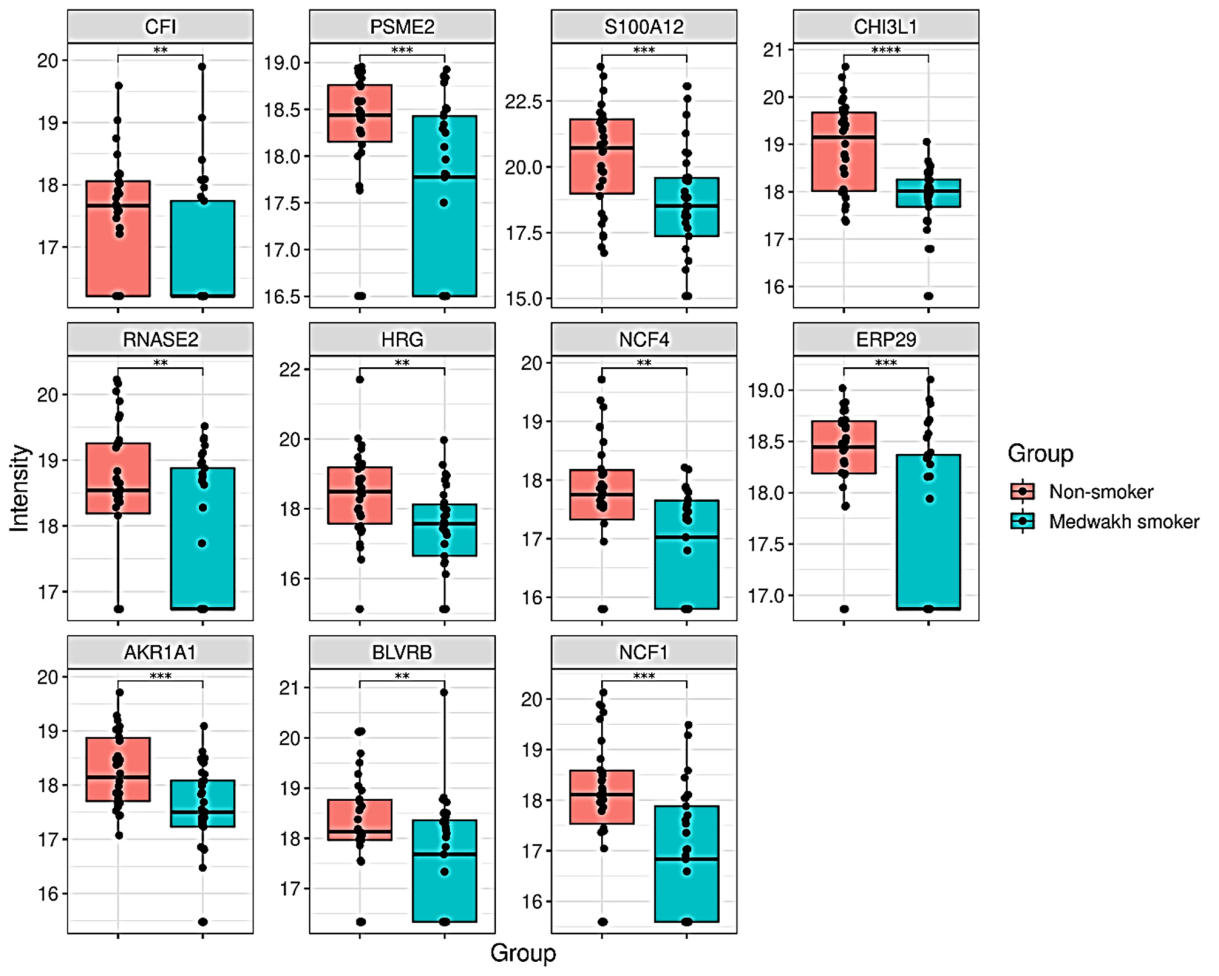
Boxplots showing log_2_ transformed protein levels involved in immune responses, inflammation, oxidative stress and antioxidant responses. Abundance of CFI (Complement factor I), PSME2 (Proteasome activator complex subunit 2), S100A12 (Protein S100-A12), CHI3L1 (Chitinase-3-like protein 1), RNASE2 (Non-secretory ribonuclease), HRG (Heme transporter HRG1), NCF 4 (Neutrophil cytosol factor 4), ERP 29 (Endoplasmic reticulum resident protein 29), AKR1A1 (Aldo-keto reductase family 1 member A1), BLVRB (Flavin reductase (NADPH) and NCF1 (Neutrophil cytosol factor 1) subdivided by medwakh smoking and non-smoking groups

The current study highlights several important findings impacted by medwakh smoking. Firstly, salivary levels of certain key immune mediators were found to be significantly altered (less abundant) in medwakh smokers compared to non-smokers, such as complement factor I (CFI) [14], proteasome activator complex subunit 2 (PSME2), calcium-binding protein S100A12, non-enzymatic chitinase-3 like-protein-1 (CHI3L1), histidine-rich glycoprotein (HRG) and non-secretory ribonuclease (RNASE2). In support of this observation, Reactome pathway enrichment analysis identified neutrophil degranulation, innate immune system, interleukin-12 signaling, and immune system as the top enriched pathways in our data. While some of these protein perturbances are observed in cigarette smokers (CHI3L1 [15], PSME2 [16], S100A12 [17] and HRG [18]), the dysregulated levels of CFI, and RNASE2 are specific to medwakh smokers (Fig. 2).

Secondly, the present study noted significant alterations in oxidative stress and antioxidant regulatory proteins in medwakh smokers, like lower abundance in neutrophil cytosol factor 4 (NCF4), endoplasmic reticulum protein 29 (ERP 29), aldo-keto reductase family 1 (AKR1), flavin reductases (NADPH BLVRB), and putative neutrophil cytosol factor 1 (NCF1). The decreased abundance of ERP29, AKR1, and flavin reductases may have negative implications on reducing oxidative burden indicating a potential imbalance between oxidative stress and antioxidant defense mechanisms (Fig. 2). This was again supported by the pathway enrichment data wherein “response to stress” and “response to chemical stress” pathways were significantly enriched.

**Fig. 3:**
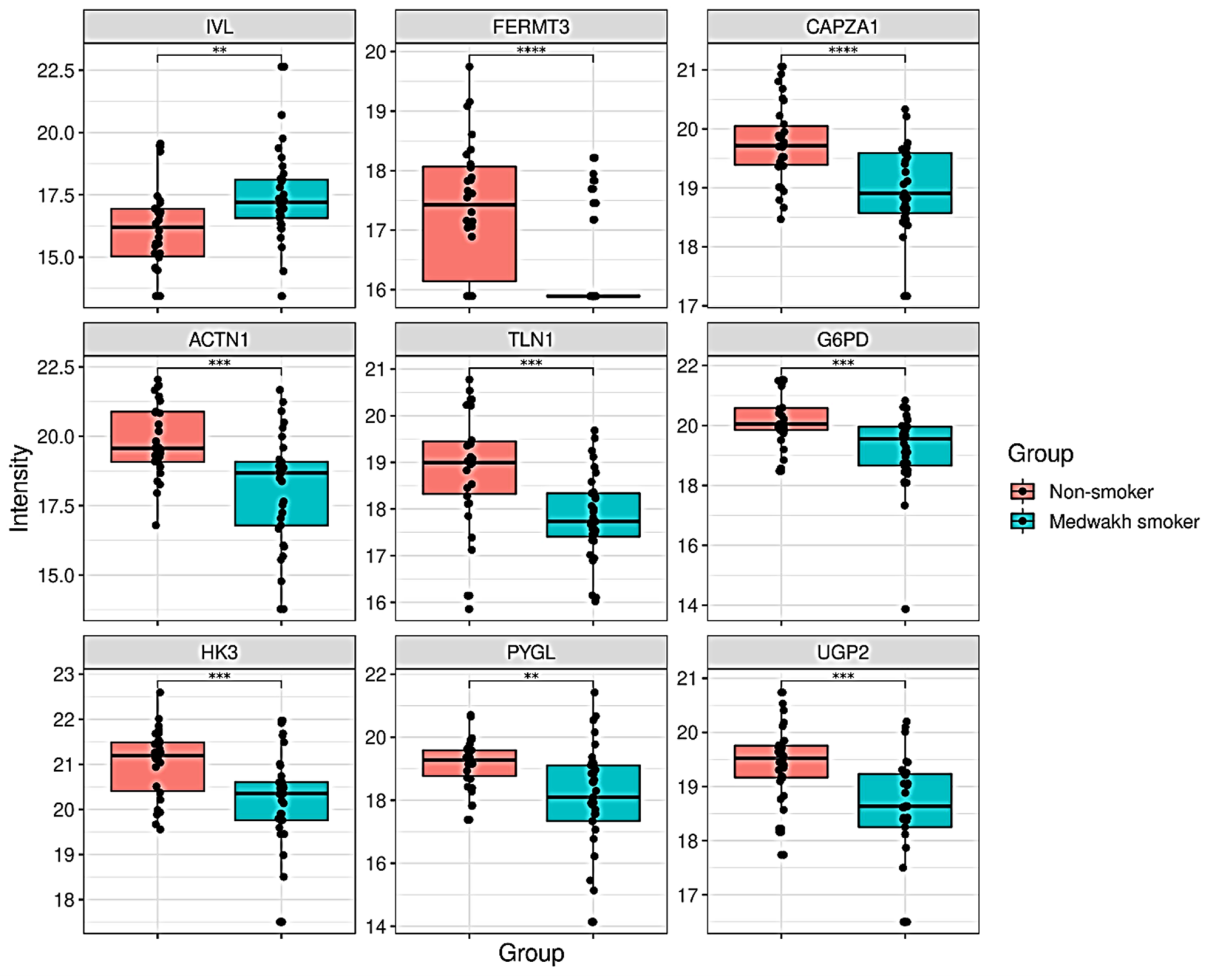
Boxplots showing log_2_ transformed proteins involved in cell differentiation, anchorage, adhesion, and various metabolic processes. Abundance of IVL (Involucrin), FERMT3 (FERM Domain Containing Kindlin 3), CAPZA1 (F-actin-capping protein subunit alpha-1), ACTN1 (Alpha-actinin-1), TLN1 (Talin-1), G6PD (Glucose-6-phosphate 1-dehydrogenase), HK3 (Hexokinase-3), PYGL (Glycogen phosphorylase, liver form) and UGP2 (UTP–glucose-1-phosphate uridylyltransferase) subdivided by medwakh smoking and non-smoking groups

Additionally, an increased abundance of involucrin (IVL), a protein associated with squamous cell differentiation, was observed in medwakh smokers compared to non-smokers. This condition is a common pathological alteration observed in habitual smokers. A rise in abundance of involucrin is reported to result in compromised epidermal permeability barrier [19] eventually leading to increased prevalence of certain cutaneous disorders (Fig. 3). Furthermore, medwakh smoking caused alterations in proteins associated with cellular dynamics including cell anchorage, adhesion and cytoskeletal structure, with all these identified among the top most enriched terms by ClusterProfiler. Tobacco smoking is reported to cause similar alterations in genes and proteins related to metabolism and remodeling of the extracellular matrix [20]. Dysregulated protein identities related to cytoskeletal and extracellular matrix components were significantly less abundant in medwakh smokers, including FERMT3, CAPZA1, ACTN1, and TLN1. These alterations are like those observed in cigarette smokers, where extracellular matrix remodeling can lead to epithelial-mesenchymal transition [21]. Downregulation of CAPZA and Talin-1 levels, associated with hemodynamic stress [22] and altered cell adhesion [23] respectively, further supports these findings. The current study further revealed alterations in the abundance of various enzymes involved in crucial metabolic pathways in medwakh smokers. Notable changes were observed in the abundance of enzymes such as G6PD, HK3, PYGL, and UGP2 (Fig. 3). These findings shed light on the pathological changes in squamous cell differentiation, cytoskeletal proteins, extracellular matrix remodeling, and cell metabolism associated with medwakh smoking.

The over-representation analysis (ORA) further strengthens these findings. The ORA highlighted significant alterations in MAPK and ERK signaling cascade, pentose phosphate pathway (PPP), cytoskeletal organization, cellular dynamics, oxidative stress, and immune response alterations. These pathways align closely with the proteomic data, linking the study’s key findings on squamous cell differentiation, oxidative stress, and alterations in immune mediators. Notably, the PPP regulates oxidative stress by producing NADPH, supporting cellular antioxidant needs in a cell-specific manner. The alterations in MAPK and ERK signaling cascade further underscore the impact of oxidative stress responses contributing to cellular damage and apoptosis. Prior studies show acute tobacco exposure activates ERK and p38 MAPK pathways leading to cell death through oxidative stress-induced apoptosis (supplementary Table 2).

To be concise, these findings shed light on the diverse pathological effects of medwakh smoking in the oral cavity, encompassing immune responses, inflammation, oxidative stress, antioxidant defense, squamous cell differentiation and alterations in cell anchorage, adhesion, cytoskeletal proteins, and cell metabolism. The unique dysregulation of CFI and RNASE2 in medwakh smokers and the observed decrease in immune mediators and alterations in oxidative stress-related proteins highlight potential health risks.

### Proteomic analysis revealed dysregulation in metabolic, cell adhesion, and cytoskeletal-associated pathways in medwakh smokers

**Fig. 4:**
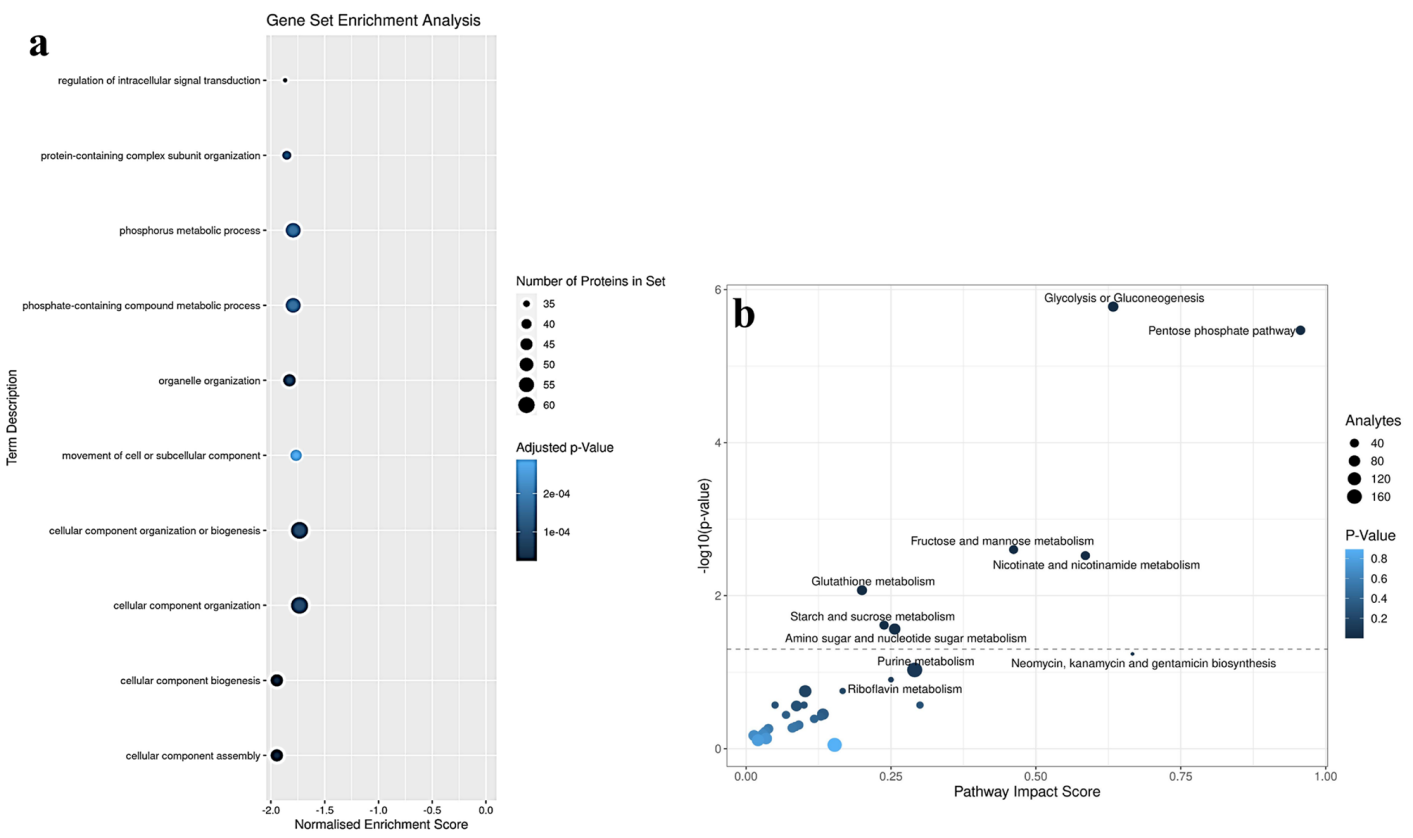
(**a**) Over-representation analysis revealed 10 significant pathways. The dots are coded based on the p-value for the enrichment. The dot size denotes the number of significant proteins in each pathway. (**b**) Joint pathway analysis of top 37 differential metabolites and 131 proteins derived from medwakh smokers in comparison to non-smoking controls. Top enriched pathways included glycolysis or gluconeogenesis, pentose phosphate, fructose and mannose metabolism, nicotinate and nicotinamide metabolism, glutathione metabolism, starch and sucrose metabolism, and amino sugar and nucleotide sugar metabolism

ClusterProfiler (version 4.2.2) revealed the overrepresented canonical pathways of significantly altered proteins in medwakh smokers compared to non-smoking controls. These include pathways related to mitochondrial damage and oxidative burst as evident from the altered expression of HEBP2 and CYFIP Related Rac1 Interactor B, the key players in the collapse of mitochondrial membrane potential before necrotic cell death and oxidative stress responses [24, 25]. The HEBP2 enhances outer and inner mitochondrial membrane permeabilization, especially under conditions of oxidative stress. Other major pathways as revealed in the enrichment analysis are related to cell adhesion, migration, differentiation, and proliferation. These pathways include the expression of proteins such as talins, kindlin-3, filamin A, etc. which are cytoskeletal proteins that are involved in the connections of major cytoskeletal structures to the plasma membrane. These proteins play a significant role in the assembly of actin filaments to membrane glycoproteins thereby contributing to remodeling of the cytoskeletal network to effect changes in cell shape and migration following various stress responses (Fig. 4a).

### Joint pathway analysis

To further investigate the impact of medwakh smoking, joint metabolic and proteomics pathways were analyzed using MetaboAnalyst 5.0 version. The 37 differentially expressed metabolites from our prior investigation and 131 differentially expressed proteins were submitted to metaboAnalyst for integration analysis. According to canonical pathway analysis, significantly altered 31 joint pathways among the medwakh smokers were identified and the most relevant ones include glycolysis or gluconeogenesis (*p* = 1.6714e-06), pentose phosphate (*p* = 3.4024e-06), fructose and mannose metabolism (*p* = 0.0025), nicotinate and nicotinamide metabolism (*p* = 0.0029982), glutathione metabolism (*p* = 0.0084934), starch and sucrose metabolism (*p* = 0.024299), amino sugar and nucleotide sugar metabolism (*p* = 0.027314), purine metabolosim (*p* = 0.093428) and pyramidine metabolism (*p* = 0.17742) (Tables 3 and Fig. 4b).

**Table 3:** Joint analysis pathways of differential proteins and metabolites

No	Pathway name	Hits	*p* value	Impact
1	Glycolysis or Gluconeogenesis	8	1.67E-06	0.63333
2	Pentose phosphate pathway	7	3.40E-06	0.95652
3	Fructose and mannose metabolism	4	0.0025	0.46154
4	Nicotinate and nicotinamide metabolism	4	0.0029982	0.58537
5	Glutathione metabolism	4	0.0084934	0.2
6	Starch and sucrose metabolism	3	0.024299	0.2381
7	Amino sugar and nucleotide sugar metabolism	4	0.027314	0.25641
9	Purine metabolism	5	0.093428	0.29091
10	Pyrimidine metabolism	3	0.17742	0.10204

### Medwakh smoking influences salivary cytokine levels

**Fig. 5:**
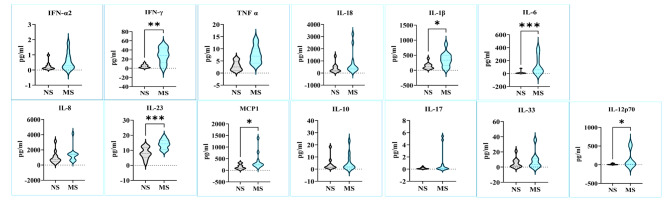
Levels of cytokines and chemokine (pg/ml) detected in saliva of medwakh smokers (MS) in comparison with non-smokers (NS)

The performance and survival of immune cells is under strict redox control, influenced by levels of intracellular and extracellular ROS/RNS species [26]. Results from the current study suggests that medwakh smoking may disrupt the delicate balance between oxidative stress and antioxidant defenses, leading to reduced levels of key immune regulators. The findings from the proteomics data were further validated by exploring expression of salivary immune response evaluation panel in medwakh smokers and compared with non-smokers. Out of the 13 cytokines measured in saliva, the levels of Th1 cytokines, (IL-1β (*p* < 0.05), IL-12p70 (*p* < 0.05), IL 23 (*p* < 0.001), IFN-γ (*p* < 0.01)), Th2 cytokine, IL-6 (*p* < 0.001) and the chemokine MCP-1 (*p* < 0.05) were significantly high in medwakh smokers compared to non-smoking controls (Fig. 5). The least abundant salivary cytokine was IFN-γ in non-smokers and in a vast majority of medwakh smokers.

## Discussion

Tobacco smoking significantly impacts the structural, functional, and molecular characteristics of airway epithelial cells, leading to oxidative stress, release of proinflammatory cytokines, impaired ciliary function, dysregulated expression and secretion of mucins, and squamous cell differentiation [25]. These variables are key biomarkers of harm, vital for monitoring the adverse health effects of tobacco smoke [25]. Although the acute effects of tobacco usage are well-documented in cigarette smokers, similar impacts in medwakh smokers remain largely unexplored. The current study addresses this gap by focusing on two primary objectives: (a) to investigate dysregulations in salivary protein profiles focusing on markers of immunity and inflammation along with cellular dynamics and oxidative stress parameters; and (b) to validate changes in salivary immune responses by exploring a detailed salivary cytokine profile among medwakh smokers. This work aims to provide insights into the health implications of medwakh smoking by comparing its effects with those seen in cigarette smoking.

Our findings indicate that Medwakh smoking significantly affects immune responses and inflammatory status in the oral cavity as reflected in the altered abundance of various proteins. For instance, increased abundance of IGLL5 and IGHV1 levels are indicative of immune cell infiltration [27] and immune response initiation [28] alongside complement activation. Conversely, the reduced levels of PSME2, S100A12, and HRG implies lung dysfunction, altered immunity [17], and poor pathogen clearance. Notably, S100A12, a multifunctional protein implicated in smoking-related pathologies, allergic asthma, and periodontal diseases, plays a critical role in regulating immune responses, chemotaxis, intracellular signaling, and oxidative stress. Likewise, CHI3L1 levels, which increases in response to lung inflammation, cigarette smoke exposure, and infections, contributes to inflammatory processes including, T-helper cell type 2 (Th2) responses and IL-13-induced inflammation.

Further in our investigation, several novel proteins related to inflammatory pathways such as CFI, RNASE2 and HRG proteins were identified in medwakh smokers. CFI plays an important role in regulating the immune response system, was significantly less abundant in medwakh smokers. Reduced CFI levels heighten vulnerability to recurrent infections, including infections of the upper respiratory tract, pneumonia, and life-threatening conditions such as meningitis, and sepsis [17]. Both CFI and RNASE2 could serve as potential markers for medwakh-associated respiratory damage. Similarly, HRG’s role in immune complex and pathogen clearance and acute phase reactions such as chemotaxis, cell adhesion, angiogenesis, coagulation, and fibrinolysis processes underscores their unique impact on medwakh smokers [18, 29], with no reported associations to tobacco smokers.

The study also found that changes observed in the proteome of key immune proteins correlated with the pro-inflammatory cytokine profile in medwakh smokers, mirroring those in chronic cigarette smokers [30]. Higher levels of IL-6 and IL-1β suggest mucosal damage and recruitment of inflammatory cells in the oral cavity. Medwakh smokers displayed higher levels of salivary IL-1β, IL-12p70, IL 23, IFN-γ, IL-6, and the chemokine MCP-1. Remarkably, IL-8 and IL-18 showed the highest level of expression in medwakh smokers. IL-8 is produced by gingival epithelial cells in response to nicotine exposure [31]. Furthermore, IL-6 expression, which promotes the proliferation of T helper and cytotoxic T cells, exacerbates the inflammatory response. IL-6, produced by T cells, B cells, and macrophages, not only stimulates B cell proliferation and immunoglobulin differentiation but also contributes to tissue pathologies associated with inflammation, indicating a complex interplay of proinflammatory cytokines in medwakh smoking-induced immune dysregulation.

Another key finding of this study relates to redox homeostasis alterations in medwak smokers. The levels of NCF4, ERP 29, AKRA1, NADPH, and NCF1B, were less abundant in medwakh users. NCF-4 plays a pivotal role in the production of ROS, maintenance of oxidative stress, by transporting electrons from NADPH to molecular oxygen, and generating reactive oxidant intermediates [32]. It has roles in host defense mechanisms. ER 29 is yet another important candidate protein that helps in managing oxidative bursts. ER 29 attenuates tobacco smoke-induced ER stress and improves cell viability. The present study observed less abundance for ER 29 in medwakh smokers aiding risk in the development of ER stress resulting from mismanagement of protein unfolding and processing by the ER [33]. Similarly, aldo-keto reductases (AKR1) and flavin reductases were also less abundant in medwakh smokers. These proteins have detoxifying roles that help in reducing a range of toxic aldehydes [34] and peroxides [35]. Additionally, Neutrophil cytosolic factor 1 (NCF1), crucial for formation of oxidative stress intermediates and host defense is also modulated significantly in medwakh smokers. These findings highlight the impact of medwakh smoking in disrupting the redox balance and its consequences on anti-oxidant defenses in agreement with our prevosuly published metabolomics data [6].

In the current study, we further highlight the impact of Medwakh smoking on altering the cellular dynamics specifically proteins involved in cell anchorage and adhesion (FERMT3, CAPZA2, ACTN1, TLN1). The altered protein profile were largely similar to cigarette smokers. For instance, FERMT3 and CAPZ are markedly downregulated in medwakh smokers and lung tissues of cigarette smokers. CAPZ, is an F-actin capping protein, that induces hemodynamic stress in the cardiac muscles [21]^,^ [22]. Down-regulation of CapZ increases cardiac myofilament calcium sensitivity, which in turn inhibits myofilament-associated protein kinases. We observed downregulation of Talin-1 (TLN) in medwakh smokers. Downregulation of TLN is reported to cause coronary artery disease development (CAD) [23]. On the contrary, an increased abundance of the protein involucrin was observed in medwakh smokers, suggestive of a pathologic alteration in the airway epithelium closely similar to habitual smokers with COPD [36]^,^ [25].

Emerging evidence suggests that tobacco smoking leads to metabolic reprogramming [37]. For example, proteomic studies of lung epithelial cells exposed to cigarette smoke revealed an abnormality in oxidative phosphorylation and altered abundance of various enzymes of the tricarboxylic acid cycle. Similarly, our study found dysregulated glucose-6-phosphate dehydrogenase (G6PD) and various other enzymes in medwakh smokers indicative of metabolic dysfunction and subsequent onset of oxidative stress [38]. Being a classical oxidoreductase, G6PD is critical to the maintenance of the NADPH pool and redox homeostasis. In addition to altering the redox equilibrium, G6PD dysregulation/deficiency leads to defective cell growth and signaling, and infection susceptibility. Additionally, we discovered lower levels of the proteins involved in glycogen synthesis (GLYG1 and UGP2), glycogen catabolism (PYGL), and hexokinases (HK3) in medwakh smokers, but their levels were unaffected in non-smokers.

Furthermore, proteomic analysis revealed alterations in key signaling pathways associated with mitochondrial damage, oxidative burst, cell adhesion, and cellular dynamics. Among the top 10 over-represented pathways by integrated pathway analysis, 5 were related to dysregulated protein biosynthesis highlighted by perturbations in glutathione metabolism, pentose phosphate, nitrogen metabolism, D-glutamine and D-glutamate metabolism, and ascorbate and aldarate metabolism. Among these dysregulated pathways, the glutathione metabolism pathway has the highest relevance to tobacco exposure. Previous studies on cigarette smokers reported a similar trend in the depletion of total glutathione (GSH + GSSG) in the airways eventually culminating in COPD [39]. In vitro and in vivo studies further confirmed this through exposure to the vapor phase of tobacco smoke. The altered glutathione metabolism pathways as observed in the present study further supported the heightened oxidative stress responses and the impaired antioxidant defense mechanisms in medwakh smokers [6]. Such responses may be the plausible reason that leads to perturbations in cellular metabolic processes in medwakh smokers.

Our proteomics investigation also suggests a potential link between medwakh smoking and alterations in purine nucleotide biosynthesis. Specifically, ADSS (adenylosuccinate synthetase isozyme 2), an enzyme involved in purine nucleotide biosynthesis, is significantly less abundant in medwakh smokers in comparison to non-smokers. Absence of ADSS can disrupt the balance of purine metabolism and impact cellular processes such as DNA replication, transcription, and energy production. Our previous metabolomics data showed a significant increase in purine degradation metabolites such as guanosine and urate in medwakh smokers. Interestingly, Toorn and colleagues reported ADSS gene inactivation in cases of tobacco smoke-induced lung adenocarcinoma [40]. Carcinogens in tobacco smoke induce mutations in key genes, promoting chromosome instability and lung tumor development. Their study highlighted selective deletion of the ADSS gene in such cases. Similarly, emerging evidence implicates dysregulation and decreased expression of RhoGAPs (ARHGAP1) [41], B4GAT1 [42], and FGA [43] in various malignancies, including cancer. The decreased abundance of these proteins in medwakh smokers suggests a comparable risk. Furthermore, the notably reduced presence of members of the oxidoreductase family (NQO2), enzymes involved in hormonal metabolism (SULT1A1), cell growth and differentiation (TSN), and those associated with cellular protein degradation (UBA1) appears to be unique to medwakh smokers. These findings shed light on potential molecular mechanisms underlying the adverse health effects associated with medwakh smoking, emphasizing the need for further research to elucidate its specific risks and implications.

## Conclusion

Our findings provide valuable insights on potential perturbations in the abundance of various key proteins, cytokines, and signaling pathways linked to medwakh smoking. We highlighted COPD-associated changes in medwakh smokers, revealing four major alterations at the protein level: such as altered immune responses leading to elevated inflammation, increased oxidation and defective antioxidant responses, dysregulated energy metabolism, and cellular alterations related to cell adhesion, migration, differentiation, and proliferation. Medwakh smoking and cigarette smoking exhibit both overlapping and distinct protein perturbations. It is interesting to note that protein dysregulations caused by medwakh smoking such as CHI3L1, PSME2, S100A12, and HRG, as well as pathways involving extracellular matrix remodeling, neutrophil degranulation, and oxidative stress are similar to cigarette smokers. However, alterations in levels of CFI and RNASE2, are unique to medwakh smoking. Both forms of smoking affect cellular dynamics, with medwakh smoking specifically affecting FERMT3, CAPZA1, ACTN1, and TLN1, the key proteins involved in cell anchorage and adhesion, mirroring similar changes observed in cigarette smokers. While metabolic reprogramming and oxidative stress are hallmarks of cigarette smoke exposure, medwakh smoking uniquely perturbs G6PD and related pathways, emphasizing its specific metabolic consequences. Although the study draws potential associations of salivary proteome alterations in young, medwakh users with indications of chronic lung diseases like COPD reported in cigarette smokers, it is important to acknowledge that these associations may require further validation and exploration in larger and more diverse populations. Given the demographic variations such as younger age and differing smoking patterns among medwakh smokers, drawing a direct connection to COPD may not be entirely accurate. It is also important to note that our study focuses on a specific population. Hence caution is warranted in generalizing these results to broader population. In addition, the observed changes in protein abundance, are primarily intracellular proteins like IVL suggesting an increased cell lysis in the oral cavity of medwakh smokers. The cross-sectional nature of our study further warrants the possibility of unaccounted confounding factors as the study did not follow the participants over time. The long-term effects of medwakh smoking on the salivary proteome and its progression to chronic diseases require additional investigation. Despite these limitations, our study is the first of its kind to investigate the salivary proteomic signature of medwakh smokers, offering novel insights into the biological effects unique to this smoking method. The findings further underscore the need for longitudinal studies in a broader population to further validate these initial observations and its association with COPD and oral cancers.

## Electronic supplementary material

Below is the link to the electronic supplementary material.

Supplementary Material 1: Data Quality. a) Distribution of the number of missed cleavages per peptide shows good digestion efficiency b) Distribution of peptide charge states centers on 2 and 3+ species as expected c) Comparison of protein LFQ abundance distributions by sample shows comparable protein loading throughout the sample injections

Supplementary Material 2: Table S1: Protein identification table generated by Maxquant

Supplementary Material 3: Table S2: Results of the over-representation analysis

## Data Availability

The dataset(s) supporting the conclusions of this article are available in the ProteomeXchange repository, via with identifier PXD045901; http://www.ebi.ac.uk/pride; Username: reviewer_pxd045901@ebi.ac.uk and Password: tLMXXceM.

## Abbreviations

ATP: Alternative tobacco products
ROS: Reactive oxygen species
COPD: Chronic obstructive pulmonary disease
CAN: Acetonitrile
DTT: Dithiothreitol
TFA: Trifluoroacetic acid
FA: Formic acid
CFI: Complement factor I
PSME2: Proteasome activator complex subunit 2
CHI3L1: Non-enzymatic chitinase-3 like-protein-1
HRG: Histidine-rich glycoprotein
RNASE2: Non-secretory ribonuclease
NCF4: Neutrophil cytosol factor 4
ERP 29: Endoplasmic reticulum protein 29
AKR1: Aldo-keto reductase family 1
NADPH BLVRB: Flavin reductases
NCF: Putative neutrophil cytosol factor 1
HPLC- MS/MS: High-performance liquid chromatography-tandem mass spectrometry
DDA: Data-dependent acquisition
