# Supplementary material for: Medwakh smoking induces alterations in salivary proteins and cytokine expression: a clinical exploratory proteomics investigation
Source: Clin Proteomics. 2025 Jan 17;22:2. doi: 10.1186/s12014-024-09520-6 (PMC11740365; doi:10.1186/s12014-024-09520-6)
Supplement: Supplementary file 1 — Supplementary Material 1: Data Quality. a) Distribution of the number of missed cleavages per peptide shows good digestion efficiency b) Distribution of peptide charge states centers on 2 and 3+ species as expected c) Comparison of protein LFQ abundance distributions by sample shows comparable protein loading throughout the sample injections [file 12014_2024_9520_MOESM1_ESM.docx]

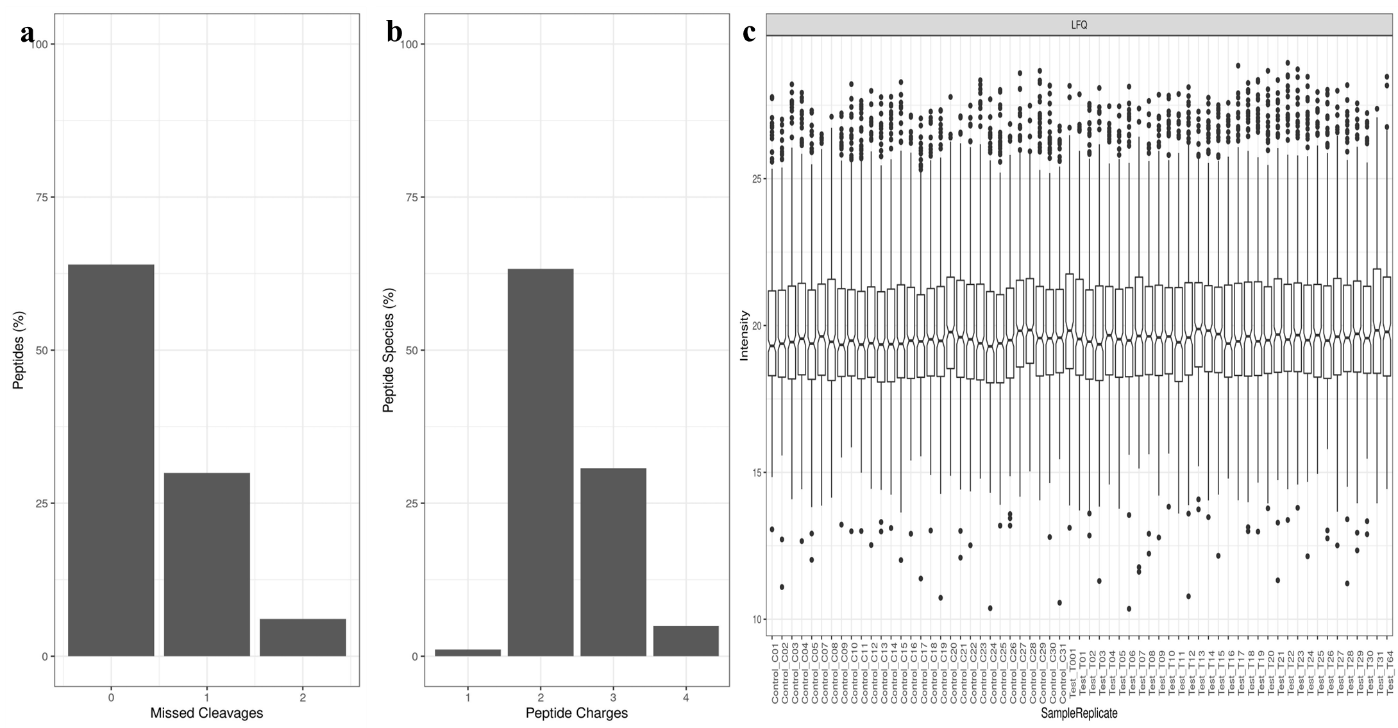


**Supplementary Figure 1:** Data Quality. a) Distribution of the number of missed cleavages per peptide shows good digestion efficiency b) Distribution of peptide charge states centers on 2 and 3+ species as expected c) Comparison of protein LFQ abundance distributions by sample shows comparable protein loading throughout the sample injections
